# In vivo assessment of endothelial permeability of coronary lesions with variable degree of stenosis using an albumin-binding MR probe

**DOI:** 10.1007/s10554-021-02293-1

**Published:** 2021-07-10

**Authors:** Leif-Christopher Engel, Ulf Landmesser, Youssef S. Abdelwahed, Kevin Gigengack, Thomas Wurster, Costantia Manes, Carsten Skurk, Alexander Lauten, Andreas Schuster, Michel Noutsias, Bernd Hamm, Rene M. Botnar, Boris Bigalke, Marcus R. Makowski

**Affiliations:** 1 Department of Cardiology, German Heart Center, Munich, Germany; 2 Department of Cardiology, Charité Campus Benjamin Franklin, Universitätsmedizin Berlin, Berlin, Germany; 3 Berlin Institute of Health (BIH), Berlin, Germany; 4 Department of Cardiology and Pulmonology, German Centre for Cardiovascular Research (DZHKPartner Site), Göttingen, Germany; 5 Department of Cardiology, Royal North Shore Hospital, The Kolling Institute, Northern Clinical School, University of Sydney, 5th Floor, Acute Services Building, Reserve Road, St Leonard’s, Sydney, NSW Australia; 6 Mid-German Heart Center, Department of Internal Medicine III (KIM-III), Division of Cardiology, Angiology and Intensive Medical Care, University Hospital Halle-Wittenberg, Halle (Saale), Germany; 7 Escuela de Ingeniería, Pontificia Universidad Católica de Chile, Santiago, Chile; 8 Department of Radiology, Charité - Universitätsmedizin Berlin, Berlin, Germany; 9 Department of Radiology, Klinikum Rechts Der Isar, TU München, München, Germany; 10 Division of Imaging Sciences and Biomedical Engineering, King’s College London, London, UK

**Keywords:** Endothelial damage, Stenosis, Molecular MRI, QCA, Target-specific MR probe

## Abstract

MR imaging with an albumin-binding probe enables the visualization of endothelial permeability and damage in the arterial system. *The goal of this study was to compare signal enhancement of lesions with different grades of stenosis segments on molecular CMR in combination with the albumin-binding probe gadofosveset.* This prospective clinical study included patients with symptoms suggestive of coronary artery disease (CAD). Patients underwent gadofosveset-enhanced cardiovascular magnetic resonance (CMR) imaging and x-ray angiography (QCA) within 24 h. CMR imaging was performed prior to and 24 h following the administration of gadofosveset. Contrast-to-noise ratios (CNRs) between segments with different grades of stenosis were compared. Overall, *n* = 203 segments of 26 patients were included. Lesions with more than > 70% stenosis demonstrated significantly higher CNRs compared to lesions < 70% (7.6 ± 8.3 vs. 2.5 ± 4.9; *p* < 0.001). Post-stenotic segments of lesions > 70% stenosis showed significant higher signal enhancement compared to segments located upstream of these lesions (7.3 ± 8.8 vs. 2.8 ± 2.2; *p* = 0.02). No difference in signal enhancement between segments proximal and distal of lesions with stenosis greater than 50% was measured (3.3 ± 2.8 vs. 2.4 ± 2.7; *p* = 0.18). ROC analysis for the detection of lesions ≥ 70% revealed an area under the curve of 0.774 (95% CI 0.681–0.866). This study suggests that relevant coronary stenosis and their down-stream segments are associated with increased signal enhancement on Gadofosveset-enhanced CMR, suggesting a higher endothelial permeability in these lesions. An albumin-binding MR probe could represent a novel *in vivo* biomarker for the identification and characterization of these vulnerable coronary segments.

## Introduction

Endothelial permeability and damage precedes the manifestation of atherosclerosis and plays a major role in its development [1]. The measurement of flow-mediated vasodilatation of the brachial artery is the noninvasive gold standard to evaluate endothelial function [2]. However, it was shown that this technique correlates only moderately with other vascular beds including the coronaries [3]. Molecular cardiac magnetic resonance (CMR) imaging using targeted molecular probes enables the visualization of biological processes, which cannot be detected by morphological imaging approaches such as cardiac computed tomography CT [4, 5]. The albumin-binding magnetic resonance-probe (MR-probe) gadofosveset-trisodium was shown to have properties comparable to the Evan’s blue dye, which represents a surrogate marker for endothelial permeability in the arterial system [4, 5]. Currently, there is only limited data on the association between local endothelial damage or dysfunction and the degree of stenosis [6].

The goal of this study was to compare signal enhancement of lesions with different grades of stenosis segments on molecular CMR. We hypothesize that lesions with higher grade of luminal narrowing are associated with stronger signal enhancement after application of an albumin-binding MR probe, which is considered as a surrogate parameter for endothelial permeability.”

## Methods

### Study population

This study included subjects with indication for invasive catheterization based on their symptoms of coronary artery disease (CAD), such as stable chest pain and acute coronary syndrome (unstable angina; Non-ST-elevation myocardial infarction/NSTEMI). All subjects were prospectively recruited between April 2015 and June 2016. All patients underwent CMR-imaging prior to (native scan) and 24-h following the administration of gadofosveset-trisodium (gadofosveset-enhanced-scan;GE-CMR). Subsequently, invasive catheterization and quantitative coronary angiography (QCA) was performed in each patient. Exclusion criteria were hemodynamically unstable patients (cardiogenic shock, rising cardiac enzymes or malignant arrhythmias, ST-elevation-myocardial-infarction;STEMI), pregnancy, patients with renal insufficiency (creatinine clearance < 30 ml/min), a history of coronary stenting, or who were not able to give their written consent (i.e. < 18 years of age, mental disorders) and common contraindication to CMR imaging (i.e. allergy to gadolinium-based contrast agents, claustrophobia, metallic – items such as cochlear implants, central nervous system aneurysm clips, pacemakers/defibrillators). All patients gave written informed consent and the study was approved by the ethics committee of Charité Universitätsmedizin Berlin for clinical investigations (**EA4/112/14**) and performed in accordance with the Declaration of Helsinki.

### Cardiac magnetic resonance imaging

All subjects underwent a CMR exam in a 3-Tesla-scanner (Magnetom Skyra, Siemens-Healthcare,Erlangen,Germany) using an 18-channel matrix coil. In all patients, CMR imaging was performed twice: Natively and 24 h following the administration of gadofosveset-trisodium (0.03 mmol/kg/body-weight), which was administered intravenously through a catheter in an antecubital vein. In this study, we used a post-contrast imaging time of around 24-h to reduce the signal from gadofosveset in the coronary lumen and to get optimal wall-to-lumen-contrast as demonstrated previously [5].

Continuous monitoring of vital signs throughout the entire CMR scan was performed with a 4-lead ECG. Patients with elevated cardiac enzymes were monitored using a CMR-compatible blood pressure monitor and blood oxygenation sensorAfter the acquisition of scout scans to identify the major structures of the heart, a cine 4-chamber-view was used to determine trigger delay and acquisition window, followed by a TI scout to determine the patient-specific inversion time to null signal from blood. For whole heart MR coronary angiography a T2-prep prepared FLASH (fast low angle shot) sequence was used including the following imaging parameters: field of view 340 × 340 mm; acquisition matrix 256 × 256; reconstruction matrix 512 × 512; acquisition slice thickness 1.3 mm; acquisition slice number 80–100; reconstruction spatial resolution 0.65 × 0.65 × 0.65 mm; repetition time/echo time 3.5 ms/1.42 ms; flip angle 20 degrees. An inversion recovery (IR) prepared 3-dimensional (3D) T1W turbo FLASH (fast low angle shot) sequence with fat suppression (FatSat) was used for whole heart coronary vessel wall imaging. Electrocardiogram – triggering and a navigator-gated free breathing technique in coronal orientation was part of each acquistion. To null blood using a region of interest to determine the most accurate value, we adjusted the patient-specific inversion time (range 270 ms to 300 ms) using the TI scout sequence. The following acquisition parameters were included: inversion time 250 ± 15 ms; field of view 340 × 340 mm; acquisition matrix 256 × 256; reconstruction matrix 512 × 512; acquisition slice thickness 1.3 mm; acquisition slice number 80–100; reconstruction spatial resolution 0.65 × 0.65 × 0.65 mm; repetition time/echo time 4.1 ms/1.3 ms; flip angle 15 degrees. The navigator gating window width was 1.5–2.5 mm. Depending on the patient’s heart rate, the data acquisition window duration time varied from 84 ms to 120 ms depending. The trigger delay and acquisition window were adjusted according to the phase with minimal motion of the right coronary artery (RCA) as determined by cine MR imaging.

### Invasive catheterization and Quantitative Coronary Angiography (QCA)

Two perpendicular projections were acquired for all coronary arteries and angiographic view with minimal foreshortening were chosen as described previously [7]. Pixel size was determined with automated distance calibration and all analyses were performed on frames demonstrating optimal luminal opacification. All QCA measurements were performed using a semi-automated edge detection system (QAngio XA 7.3,Medis,Leiden,Netherlands). The proximal and distal limits of the lesion were set manually before a semi-automated edge-detection software then delineated the lesion contours and provided the reference vessel diameter and luminal diameter at maximal obstruction. The minimal lumen diameter (MLD) was defined as the smallest luminal diameter in the segment of interest. The diameter stenosis in % was automatically calculated. Lesions were stratified into five different categories based on the extent of stenosis: 100%, 70–99%, 50–69%, 30–49%, 0–29%.

### CMR image analysis

CMR analysis, was performed using a dedicated image analysis software (Osirix-3.6.1,Geneva,Switzerland). Signal enhancement of coronary segments was determined on GE-CMR and the precontrast CMR-scan according to a 9-Segment model. Contrast-to-noise ratio (CNR) was defined as the difference in signal enhancement between the coronary segment and blood divided by the background noise (SI lesion–SI blood/noise). The standard deviation of the signal enhancement in a region of interest ventrally to the patient´s chest was used to obtain the background noise [5].

### Statistics

We used the SPSS-software (IBM-SPSS-Statistics-Version24) for statistical analysis. Continuous variables were reported as mean standard deviation or median with interquartile range (25th and 75th percentiles). Nominal variables were reported as percentage or frequencies. Differences in continuous variables were calculated using the Kruskal–Wallis-test or analysis-of-variance (ANOVA), when appropriate. Nominal variables were compared among the three groups using chi-square and Fisher-exact-tests, when appropriate. A 2-tailed *p*-value < 0.05 was reported as statistically significant.

## Results

Clinical baseline characteristics (*n* = 26) are shown in the supplemental Table 1. Coronary segments, which were not correctly visible on invasive catheterization due to overlap of other vessels of foreshortening (i.e. wrong projections), were excluded. In addition, *n* = 31 segments, mainly in the distal left arterial desceding artery (LAD), left circumflex artery (LCX) or right coronary artery (RCA), had to be excluded due to insufficient image quality on CMR. Consequently, 203/234 coronary segments were available for analysis in the coronary artery scan 24 h following the administration of the albumin-binding-MR-probe.

**Table 1:** Baseline patients´ characteristics and medical treatment upon admission

	All patients (*n* = 26)
Age, y	69.2 ± 13.2
Male, *n* (%)	17 (65.4)
Weight, kg	81.2 ± 19.6
BMI, kg/m2	27.4 ± 6.8
*Risk factors*	
Hypercholesterolemia, *n* (%)	13/26 (50)
Hypertension, *n* (%)	22/26 (84.6)
Diabetes mellitus, * n* (%)	8/26 (30.8)
Smoking, *n* (%)	12/26 (46.2)
Family history of CAD, *n* (%)	5/26 (19.2)
*Laboratory findings*	
Troponin T, ng/ml	141.2 ± 380.4
CK, UI/l	147.3 ± 104.3
CK-MB, UI/l	29.4 ± 30.9
Creatinine, mg/dl	1.0 ± 0.3
C-reactive protein, mg/dl	36.9 ± 76.2
Platelets, × 109	252.2 ± 56.5
Total cholesterol, mg/dl	182.4 ± 46.8
Triglyceride, mg/dl	160.2 ± 103.7
HDL cholesterol, mg/dl	46.3 ± 14.3
LDL cholesterol, mg/dl	113.1 ± 42.9
*Medication*	
Aspirin	16/26 (61.5)
Statin	9/26 (34.6)
Beta-blocker	11/26 (42.3)
ACEI and/or ARB	16/26 (61.5)

### Invasive catheterization and quantitative coronary angiography (QCA)

The total amount of coronary segments containing a total occlusion were *n* = 8. The number of coronary segments with a stenosis of 70–99%, 50–69%, 30–49% and 0–29% were *n *= 13, *n* = 31, *n* = 83 and *n* = 68, respectively. The mean length of lesions in the stenosis-category 70–99%, 50–69%, 30–49% and 0–29% was 7.9 ± 4.4 mm, 6.4 ± 2.7 mm, 5.0 ± 3.8 mm and 3.8 ± 3.5 mm, respectively.

### Comparison of signal enhancement between segments with variable degree of stenosis on Gadofosveset-enhanced CMR

**Fig. 1:**
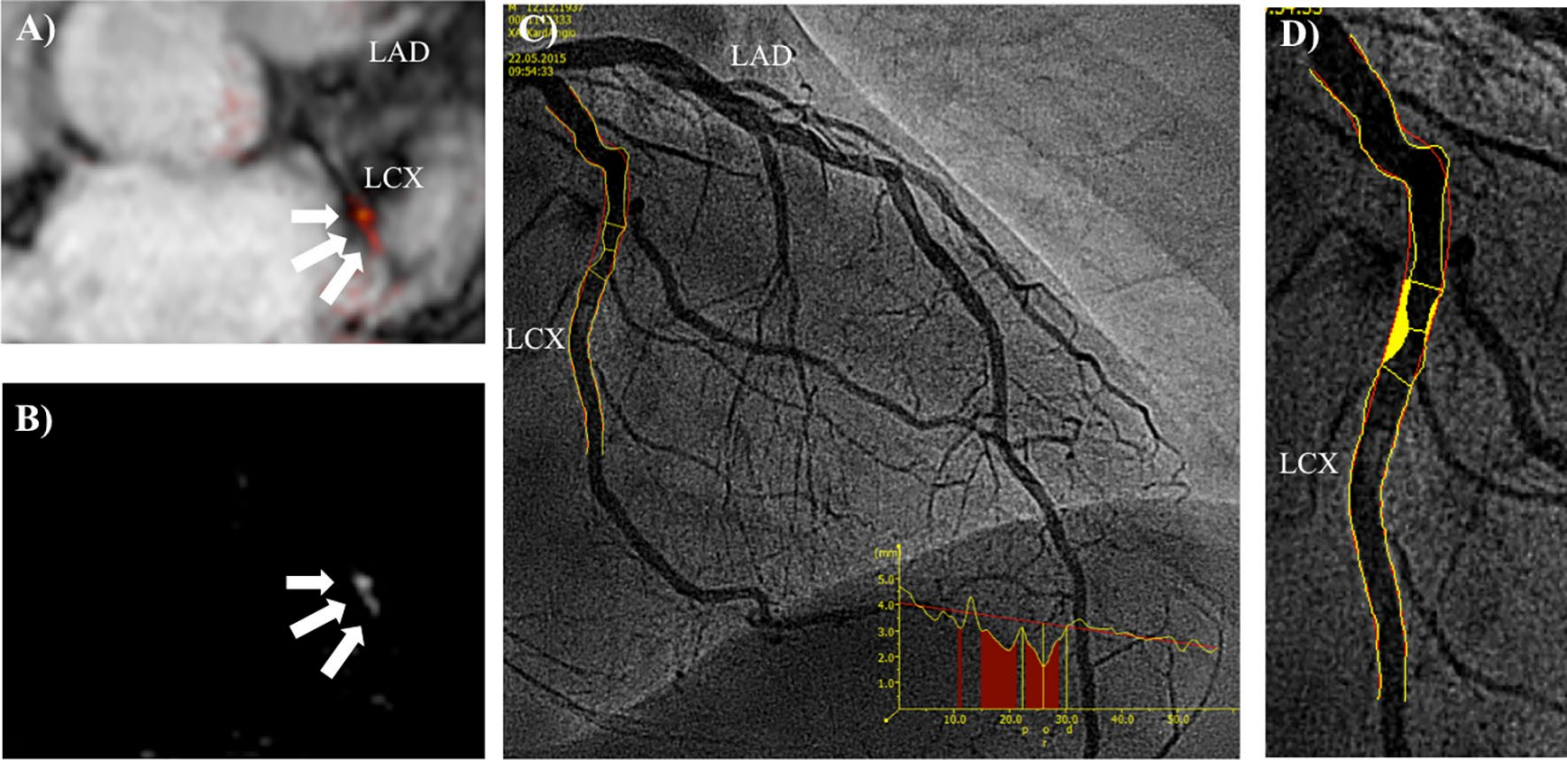
Representative images following the administration of an albumin-binding MR probe for the assessment of endothelial permeability and damage at sites of significant stenosis. To highlight the anatomical relationship between the uptake of the albumin-binding-MR probe (**b**) and morphology from the MR angiography, images were fused in a way comparable to positron emission tomography/computed tomography (**a**). Subsequent Invasive catheterization demonstrated a stenosis in the left circumflex artery (LCX) (**c**). The assessment using quantitative coronary angiography (QCA) revealed a stenosis of 73% (**d**). This sample case illustrates that CMR in combination with an albumin-binding probe is able to clearly identify coronary artery stenosis greater than 70%. Signal enhancement following application of gadofosveset-trisodium is known to be a surrogate for endothelial permeability and damage, which may be increased in severe stenosis

**Fig. 2:**
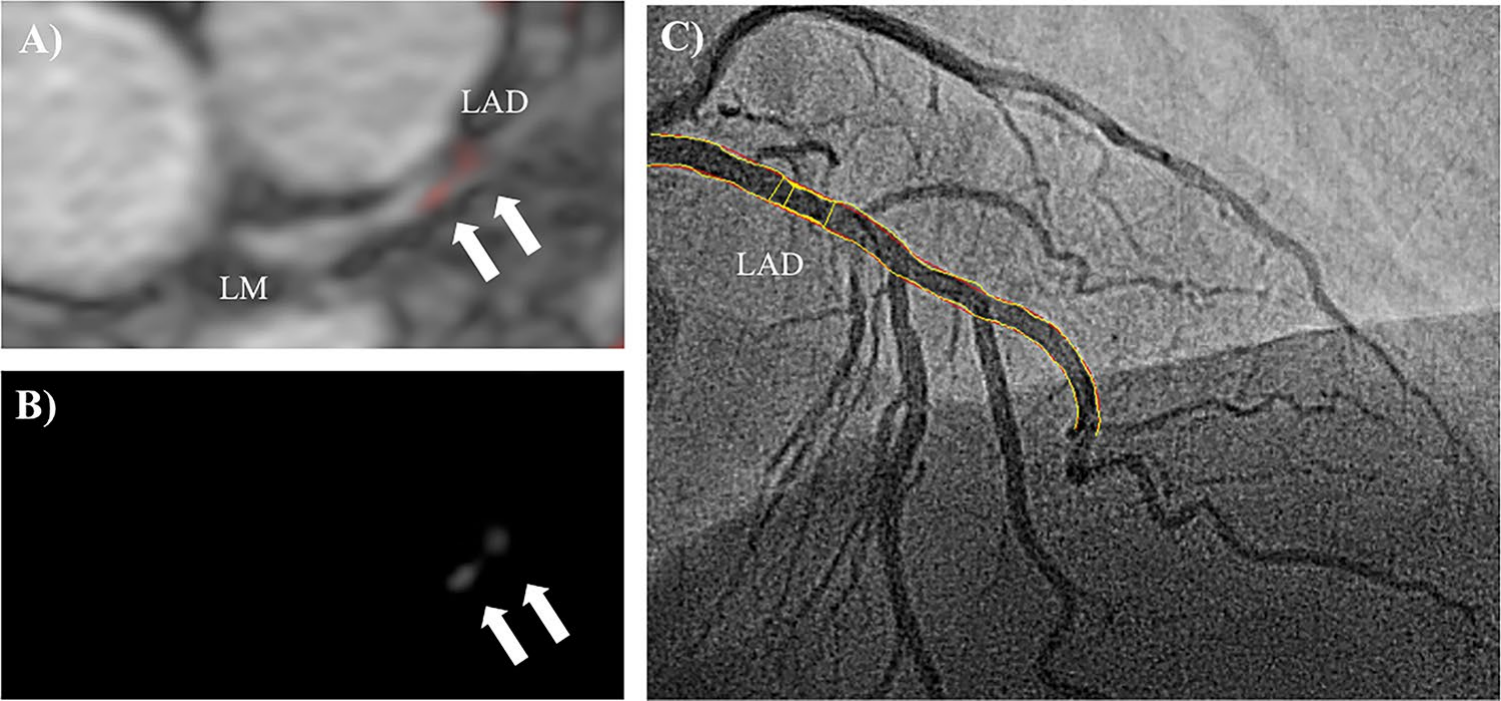
Representative images demonstrating on minimal signal enhancement at sites of non-relevant stenosis following the administration of an albumin-binding MR probe. To highlight the anatomical relationship between the uptake of the albumin-binding-MR probe (**b**) and morphology from the MR angiography, images were fused in a way comparable to positron emission tomography/computed tomography (**a**). Subsequent Invasive catheterization demonstrated only a minimal stenosis in the left arterial descending artery (LAD) (**C**)

**Fig. 3:**
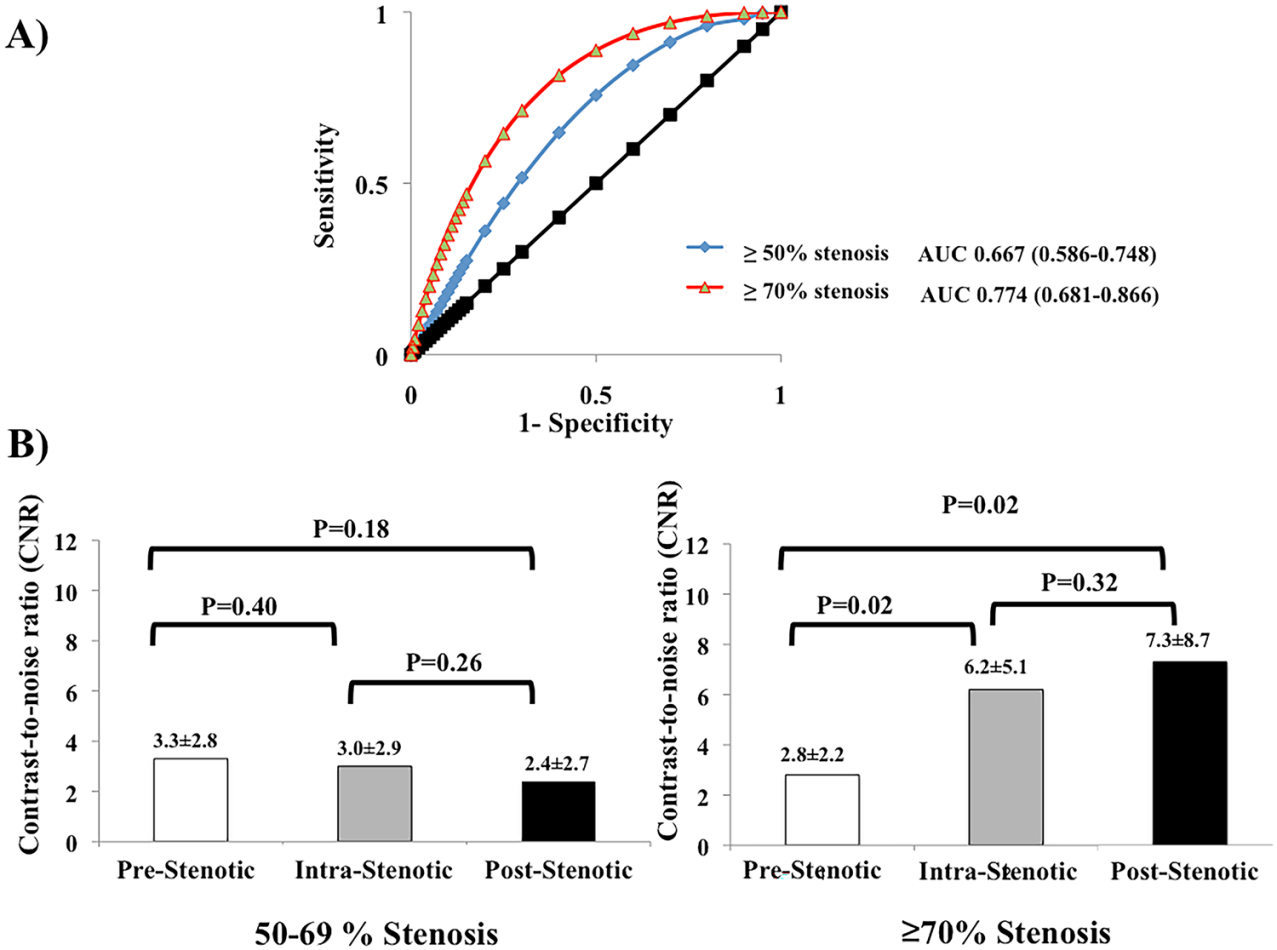
**a** ROC analysis for the detection of stenosis ≥ 50% and ≥ 70% revealed an AUC of 0.667 (95% CI 0.586–0.748) and 0.774 (95% CI 0.681–0.866) respectively. Differences between **b** prestenotic, intrastenotic (50–69% stenosis) and poststenotic segments and **c** prestenotic, intrastenotic (≥ 70% stenosis) and poststenotic segments are demonstrated

Lesions greater than 70% stenosis demonstrated significantly higher contrast-to-noise ratio (CNR) values as compared to lesions below that threshold (7.6 ± 8.31vs.2.5 ± 4.9;p < 0.001). Figure 1 demonstrates a sample where high signal enhancement at sites of lesions with greater than 70% stenosis is visible (see Fig. 1), whereas Fig. 2 shows only mild signal enhancement on Gadofosveset-enhanced CMR at sites of lesion with only minimal to mild stenosis (see Fig. 2). ROC analysis for the detection of lesions greater than 50% and greater than 70% revealed an area under the curve (AUC) of 0.667 (95% CI 0.586–0.748) and 0.774 (95% CI 0.681–0.866), respectively (see Fig. 3A). There was no difference in CNR values between segments located proximally and distally of lesions with stenosis greater than 50% (3.3 ± 2.8 vs. 2.4 ± 2.7; p = 0.18) (see Fig. 3B). However, segments located distally of lesions with greater than 70% stenosis showed significant higher signal enhancement compared to segments located proximally of these lesions (7.3 ± 8.8 vs. 2.8 ± 2.2; *p* = 0.02) (see Fig. 3C).

### Comparison of signal enhancement between the pre-contrast and Gadofosveset-enhanced CMR exam

Significant differences regarding signal enhancement between the non-contrast-enhanced baseline scan and the gadofosveset-enhanced scan (GE-CMR) were seen in all segments with a stenosis ≥ 50% (4.7 ± 6.1vs.3.0 ± 3.9; *p* = 0.03). No differences in signal enhancement between the non-contrast-enhanced baseline scan and the gadofosveset-enhanced scan (GE-CMR) were observed in all segments containing a stenosis < 50% (2.5 ± 5.3 vs. 2.5 ± 6.3; *p* = 0.487). Similarily, signal enhancement between the non-contrast-enhanced baseline scan and the gadofosveset-enhanced scan (GE-CMR) differed significantly between segments with a stenosis ≥ 70% (7.6 ± 8.3 vs. 1.9 ± 3.4; *p* = 0.003). In contrast, segments containing a stenosis < 70% (2.5 ± 4.9 vs.2.7 ± 6.0; *p* = 0.407) demonstrated equal signal enhancement between the non-contrast-enhanced baseline scan and the gadofosveset-enhanced scan (GE-CMR).

## Discussion

This study suggests that severe stenotic lesions are associated with an increase in endothelial permeability and damage. Lesions causing luminal narrowing impair endothelial function, especially in post-stenotic segments.

### Stenosis severity and Vulnerable Plaque

The measurement of brachial artery flow-mediated vasodilatation is the noninvasive gold standard to determine endothelial function and represents a surrogate parameter for predicting cardiovascular disease progression and outcome [2].

Local and low-grade systemic inflammation promotes the development of atherosclerosis. Finally, the Canakinumab Anti-inflammatory Thrombosis Outcomes Study (CANTOS) has provided strong evidence in support of the inflammation hypothesis [8]. In this regard, it was shown recently that the C-reactive protein to albumin ratio (CAR), which is seen as a novel marker of inflammation, had a very strong diagnostic value in detecting significant CAD [9].

MR-imaging with an albumin-binding probe instead yields information on the presence and extent of endothelial permeability and damage rather than inflammation, which plays a also major role in the development of atherosclerosis and plaque formation [1]. Additionally, it has been suggested that signal enhancement after application of an albumin-binding MR probe is a surrogate marker for the detection of high-risk coronary plaques [4]. Until recently it was widely believed that these plaques were only associated with mainly mild to moderate luminal narrowing [7]. However a study suggested later on that it is twice as likely for a lesion to be a thin-cap fibroatheroma in severe stenosis that in a non-severe stenosis [10], supporting our assumption that signal enhancement after application of an albumin-binding MR-probe is a not only a surrogate marker for the detection of vulnerable plaques but also of severe stenosis. For, plaque growth is associated with intraplaque hypoxemia leading to the proliferation of fragile neovessels with “leaky” endothelium [4, 11], which is the target of the albumin-binding MR-probe used in this study. Inward expansion in case of stenosis may have the same effect on neoangiogenesis as outward expansion of a plaque (i.e. positive remodeling) which is a feature of high-risk plaques [11]. The higher signal enhancement in post-stenotic segments suggests that the constantly reduced blood flow in post-stenotic segments results in endothelial damage, reflected by increased endothelial permeability. In line with this, Heinen et al. observed a decrease in vasodilate function as assessed by flow-mediated vasodilation down stream of significant stenosis [6].

### Clinical implication

MR imaging with an albumin-binding probe has been shown to detect and visualize vascular and endothelial damage, rather than relevant areas of stenosis, which is commonly defined as > 70% stenosis in a major coronary vessel, or > 50% stenosis in combination with fractional flow reserve ≤ 0.8 [12]. Based on our data, these sites may not only be characterized by increased endothelial permeability itself, but also may negatively affect the endothelium downstream, predisposing them for plaque formation. Therefore, the added information of MR imaging with an albumin-binding MR-probe could improve risk stratification beyond risk scores such as the SYNTAX score, for instance, which relies solely on coronary anatomy and lesion characteristics [13].

### Limitations

Only 26 patients were included, limiting the findings in this study and preventing us to include a meaningful correlation analysis of CNR and stenosis. Findings of this small feasibility study were not compared to flow-mediated vasodilatation of the brachial artery. However, previous studies demonstrated that there is strong evidence that the albumin-leakage-sign in CMR represents a surrogate marker for pathological endothelial function and endothelial permeability [4, 5]. Finally, MR-imaging with an albumin-binding probe is still associated with a relatively long scan time for the assessment of the coronary enhancement in vivo, which currently limits its applicability in a wide clinical setting. Using more advanced motion correction techniques in combination with undersampled image reconstruction (e.g. compressed sensing), this limitation may be solved in the future [14].

## Conclusion

This study suggests that high-grade stenotic coronary lesions are associated with an increase in endothelial permeability. Lesions causing luminal narrowing greater than 70% may impair endothelial function especially in post-stenotic segments. An albumin-binding MR probe could represent a novel *in vivo*-biomarker for identification and characterization of these vulnerable coronary segments.

## Abbreviations

AUC: Area under the curve
CAD: Coronary artery disease
CMR: Cardiovascular magnetic resonance imaging
CNR: Contrast-to-noise ratio
GE-CMR: Gadofosveset-enhanced cardiovascular magnetic resonance imaging
ICA: Invasive coronary angiography
MLD: Minimal lumen diameter
MR: Magnetic resonance imaging
NSTEMI: Non-ST-elevation myocardial infarction
QCA: Quantitative coronary angiography
